# Erythrodermic cutaneous T-cell lymphoma: Insights from a Kenyan perspective

**DOI:** 10.1016/j.jdin.2025.05.009

**Published:** 2025-06-16

**Authors:** Caroline Mutio, Hannah Wanyika, Daniel Zuriel, Caroline Ndarathi

**Affiliations:** a Department of Dermatology, Kenyatta National Hospital, Nairobi, Kenya; b Department of Pathology, Kenyatta National Hospital, Nairobi, Kenya

**Keywords:** cutaneous t-cell lymphoma (CTCL), erythrodermic-CTCL (E-CTCL), erythrodermic MF (eMF), epidemiology, mycosis fungoides (MF)

*To the Editor:* Primary cutaneous T-cell lymphomas (CTCL) are extranodal non-Hodgkin lymphomas primarily affecting the skin, with mycosis fungoides and Sézary syndrome comprising the majority subtypes.^1^ Erythrodermic CTCL (E-CTCL), assigned a T4 skin rating, accounts for 10% of CTCL cases and is classified into erythrodermic mycosis fungoides (eMF), Sézary syndrome, and E-CTCL, not otherwise specified.^2^ We present 13 cases of E-CTCL from Kenyatta National Hospital, highlighting distinct clinical patterns, histological variants, and treatment challenges.

Among 13 patients (11 males and 2 females), the mean age at diagnosis was 50.8 ± 17.9 years. Erythrodermic mycosis fungoides predominated in 92.3% (12/13), with one case of Sézary syndrome. Eczema was the most frequent initial diagnosis, followed by pityriasis rubra pilaris (15.3%). Histological confirmation was achieved in all cases; folliculotropic and syringotropic variants were identified in one case each. Clinically, widespread variegated scaly patches and plaques (69.2%) and ulcerated tumors (30.8%) were common. A rare variant, generalized poikilodermatous mycosis fungoides (MF) was seen in 2 patients (Fig 1), whereas hypopigmented and vitiligo-like leukoderma patches were observed in one patient (Fig 2). Palmoplantar keratoderma and leonine facies appeared in 100% and 15.4% of the cases, respectively. All patients received systemic therapy with multiagent chemotherapy used as first-line regimen in 53.8% and methotrexate (± retinoids) in 46.2%. Phototherapy (Narrow Band-UVB or Psoralen UVA) was administered to 3 patients each. Total skin electron beam therapy was performed in 2 patients, with favorable responses. Two patients succumbed to disease progression, while 11 are undergoing treatment.

**Fig 1:**
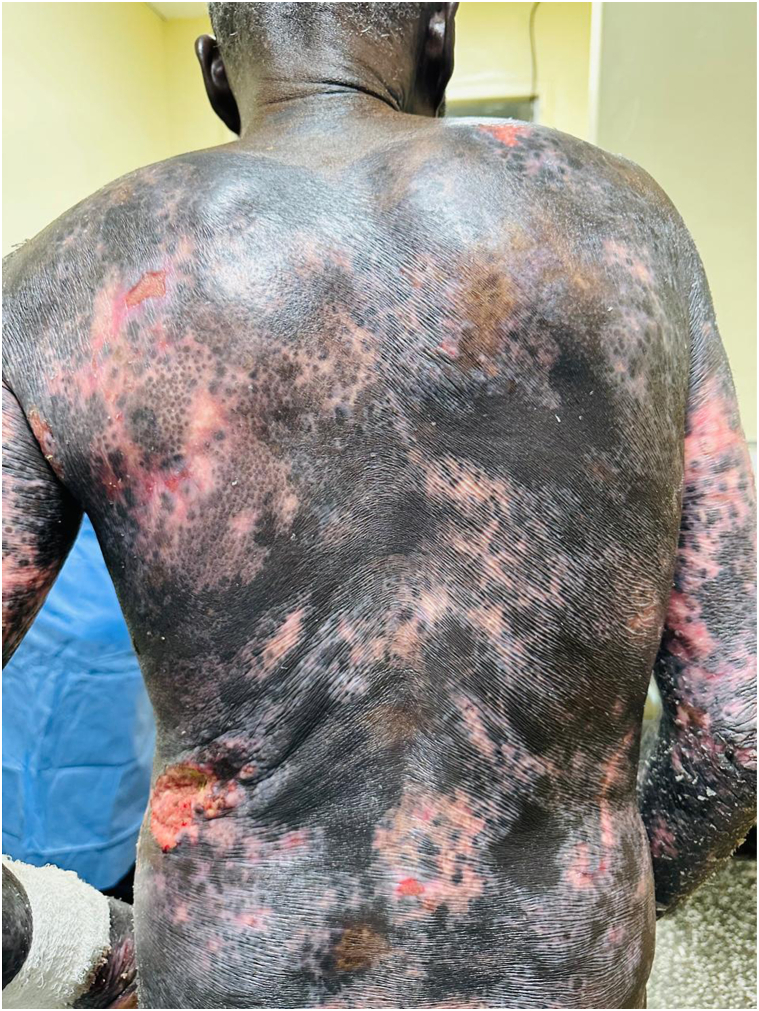
Generalized poikilodermatous mycosis fungoides presenting with diffuse mottled pigmentation, atrophic skin, and islands of sparing.

**Fig 2:**
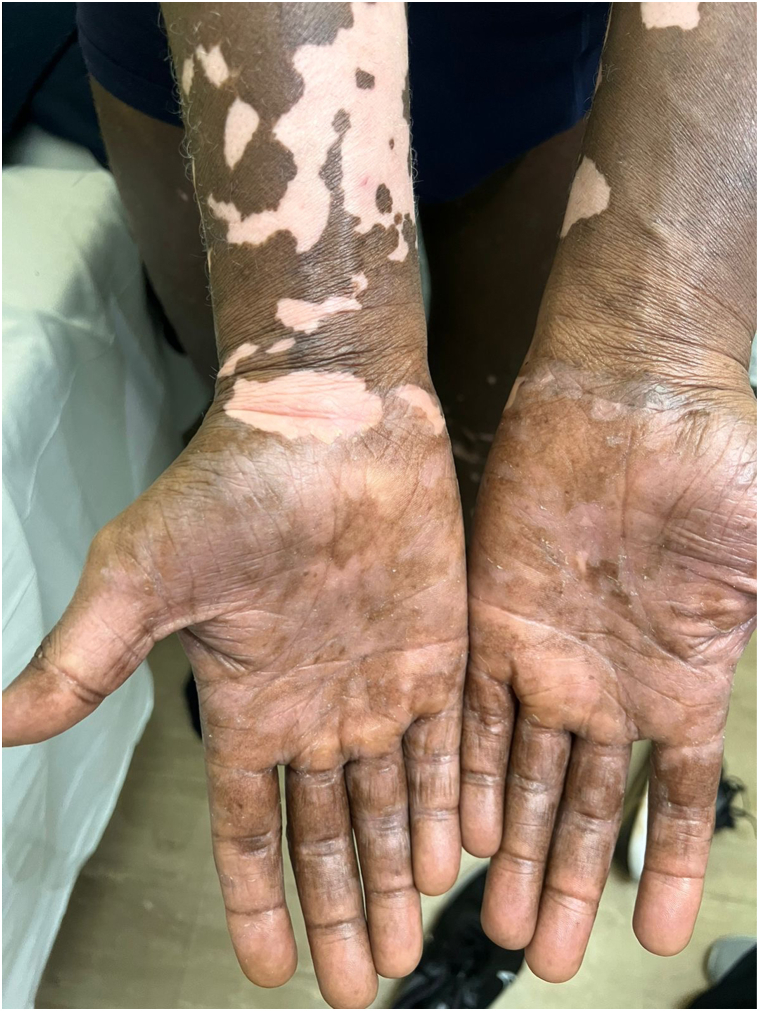
Vitiligo-like leukoderma patches noted following resolution of erythroderma.

This study emphasizes the distinct clinical profile of E-CTCL in Black populations with a younger age at onset, advanced disease at presentation, and diagnostic delays averaging 5.15 years. This pattern appears consistent with previously reported racial disparities in MF.^3^ The predominance of eMF over Sézary syndrome contrasts with Western cohorts, likely reflecting limited access to sensitive diagnostics such as flow cytometry.^2^

Occupational exposures, especially to agrochemicals, were reported in 69.2% of patients, aligning with previous evidence suggesting environmental risk factors, although we did not examine the specific herbicide or pesticide used.^4^ Universal advanced-stage disease necessitated systemic treatment, with all patients requiring a mean of 2 different systemic regimens and 3 patients receiving third-line chemotherapy; yet durable control remained limited due to lack of access to targeted biologics and radiation therapy.^5^ Many patients in this series reported experiences suggestive of psychosocial burden, including stigma from disfiguring tumors, financial strain, and emotional distress from chronic pruritus. Although these were not formally measured, these observations highlight the broader impact of E-CTCL on quality of life. These findings underscore the need for heightened clinical suspicion, innovative use of available diagnostic tools, and pragmatic treatment strategies to improve outcomes in resource-limited settings. Further studies exploring genetic and environmental risk factors are warranted.

## Conflicts of interest

None disclosed.

## References

[1] Hristov A.C., Tejasvi T., Wilcox R.A. (2023). Cutaneous T-cell lymphomas: 2023 update on diagnosis, risk-stratification, and management. Am J Hematol.

[2] Vonderheid E.C., Bernengo M.G., Burg G. (2002). Update on erythrodermic cutaneous T-cell lymphoma: Report of the international society for cutaneous lymphomas. J Am Acad Dermatol.

[3] Huang A.H., Kwatra S.G., Khanna R., Semenov Y.R., Okoye G.A., Sweren R.J. (2019). Racial disparities in the clinical presentation and prognosis of patients with mycosis fungoides. J Natl Med Assoc.

[4] Aschebrook-Kilfoy B., Cocco P., La Vecchia C. (2014). Medical history, lifestyle, family history, and occupational risk factors for mycosis fungoides and Sézary syndrome: The interlymph non-Hodgkin lymphoma subtypes project. J Natl Cancer Inst Monogr.

[5] Hughes C.F.M., Khot A., McCormack C. (2015). Lack of durable disease control with chemotherapy for mycosis fungoides and Sézary syndrome: a comparative study of systemic therapy. Blood.

